# A coronavirus disease 2019 (COVID-19) outbreak in a hospital and hospital closure: A note

**DOI:** 10.1017/ice.2020.194

**Published:** 2020-05-04

**Authors:** Rujittika Mungmunpuntipantip, Viroj Wiwanitkit

**Affiliations:** 1 26 Medical Center, Bangkok Thailand; 2 Dr DY Patil University, Pune, India; 3 Hainan Medical University, Haikou, China; 4 Chulalongkorn University, Bangkok Thailand

*To the Editor*—The coronavirus disease 2019 (COVID-19) outbreak is a global public health problem. After its occurrence in the Republic of China in December, 2019, the disease spread worldwide, occurring in >160 countries.^1^ The virus is highly contagious, and close contact in crowded places is an important contributing factor to SARS-CoV-2 transmission. Medical personal are an important at-risk population that can get COVID-19 due to close contact with patients in daily practice.^2,3^ Sporadic case reports about COVID-19 from many countries have been published.^4,5^ Hospital-acquired infection is possible, as are hospital outbreaks. Here, we present data from Thailand, the second country where the disease occurred in early January 2020.^6^

The setting is a rural district hospital in Yala province, the southernmost province of Thailand that shares an international border with Malaysia, another country where COVID-19 outbreaks occur. This small, 30-bed hospital serves local people in that rural district. The outbreak occurred on March 22, 2020, when 3 medical personnel (2 nurses and 1 physician) developed fever and COVID-19 was confirmed after they had provided regular care to local people with a history of COVID-19 contact. All 3 medical personnel are presently under respiratory isolation, but none has had lung complications. All of the other 21 medical personnel of the hospital, including 7 physicians, are under quarantine. The hospital had to be closed and no longer provides any service. According to our best knowledge, this is the first report of COVID-19 outbreak in a hospital that resulted in total disruption of hospital function.

The COVID-19 outbreak in hospitals is serious because it can result in abrupt cessation of local medical care and especially management of COVID-19 during the outbreak. Hospitals usually have protective systems in place, but a high-volume load might result in unsuccessful disease control. A good hospital infection control program during COVID-19 should be simple but strict, with aggressive procedures that might differ from routine clinical practice. Strategic planning to reduce unnecessary physical examinations, to implement universal drug distribution, or to postpone unnecessary procedures during the crisis period, is necessary.
